# Voluntary Hospitals and Local Health Authorities: What University College Hospital Is Doing

**Published:** 1915-12-11

**Authors:** 


### VOLUNTARY HOSPITALS AND LOCAL HEALTH AUTHORITIES.

### What University College Hospital is Doing.

The practical manner in which University College Hospital is co-operating with the health authorities of the contiguous Boroughs of Holborn and St. Pancras was explained by the Secretary to a representative of THE HOSPITAL in an interview this week. Although this institution has provided accommodation for 220 wounded soldiers, its recently inaugurated work in connection with the treatment of tuberculosis and child welfare in these densely populated areas is proceeding smoothly and with excellent results.

### The Tuberculosis Dispensary.

The work of the tuberculosis dispensary, which was established about a year ago, is steadily growing and increasing in usefulness. On an average there are about 100 attendances each week, but the tendency is for the weekly attendances to increase in number; this, of course, is the natural consequence of this branch of the hospital's activity becoming more widely known among the large population covered by the scheme. The dispensary serves the whole of the Borough of Holborn and three wards in St. Pancras, an area comprising a hundred and fifty thousand people. In connection with the dispensary there is a tuberculosis officer and a specially trained nurse who acts as a health visitor. Cases come up for treatment in the ordinary way and are visited in their own homes. This department forms part of a comprehensive scheme for the treatment of tuberculosis in London, and the hospital's tuberculosis officer co-operates with the Medical Officer of Health of the Boroughs of Holborn and St. Pancras and the Medical Officer of Health of the London County Council, and the scheme is officially recognised by the Local Government Board. In connection with the department are four observation beds and ten in-patient beds divided equally between males and females.

### Maternity and Child Welfare Department.

The work done by University College Hospital in connection with the treatment of tuberculosis forms a useful object-lesson in the way in which voluntary institutions can co-operate with public authorities. Certain other hospitals, notably St. Thomas's, are working on the same lines, and it is a moot point as to which of these two institutions is the pioneer in this direction. That, however, is not a question which need be discussed here; the main point is that the policy is an excellent one—a policy which, in the interests of the public health no less than voluntary hospitals, could with advantage be adopted by other institutions of this class. Whether or not St. Thomas's Hospital or University College Hospital was the first to co-operate with local authorities in the treatment of tuberculosis, it seems to be clearly established that the latter institution was the pioneer in another branch of public-health work—namely, the Maternity and Child Welfare Department. At University College Hospital this has grown out of the Obstetric Department; it is, indeed, a natural offshoot of that branch. It provides for the instruction of expectant mothers and mothers with reference to the care of infants at and after birth up to the age of eighteen months, classes being held on three days in each week. The department—like the tuberculosis dispensary—co-operates with the municipal health authorities of Holborn and St. Pancras, and health visiting is undertaken by arrangement with those bodies. The hospital provides four observation beds for ante-natal cases, and eight maternity beds for complicated cases. It is worthy of note that the hospital has recently secured a grant in aid of this department from the Local Government Board. In view of the special encouragement that is now being given to organised efforts to provide for the comfort and the health of expectant mothers, and to preserve the lives and health of infants and to promote their welfare, the work in this connection which is being carried on with such conspicuous success by University College Hospital is worthy of the greatest publicity. It is also a fact deserving to be specially mentioned that, notwithstanding the strain which the war has put upon this as upon all other institutions of the kind, this beneficent work of guarding young mothers and fostering little babies is proceeding smoothly and on lines which promise results of national importance. To emphasise what we have already said in other words, the policy of hospitals working in connection with local authorities deserves to be widely copied.

### Accommodation for Wounded Soldiers.

It is remarkable that the work to which allusion has just been made was begun in war-time, and has developed to its present important dimensions during a period when the hospital has provided beds for over two hundred wounded soldiers. University College placed at the disposal of the hospital for the duration of the war the new eugenics laboratory—which adjoins the hospital buildings—and this has been converted into a hospital of 110 beds. In addition to this, three of the hospital wards have been set apart for the accommodation of military patients. In order to do this, it has been necessary to reduce the accommodation available for ordinary patients by about a hundred beds; but, in spite of this fact, the list of civil cases awaiting admission is barely above the normal. This apparently curious phenomenon is partly capable of being explained by the fact that the number of cases seeking admission has been considerably reduced since the war began, owing to various causes, notably the increased prosperity of the working classes, which has enabled them to buy more nourishing food or to secure private instead of hospital treatment.

